# Effects of Landscape Patterns and Their Changes to Species Richness, Species Composition, and the Conservation Value of Odonates (Insecta)

**DOI:** 10.3390/insects12060478

**Published:** 2021-05-21

**Authors:** Aleš Dolný, Stanislav Ožana, Michal Burda, Filip Harabiš

**Affiliations:** 1 Department of Biology and Ecology, Faculty of Science, University of Ostrava, Chittussiho 10, CZ-710 00 Ostrava, Czech Republic; 2 Institute for Research and Applications of Fuzzy Modeling, University of Ostrava, 30. dubna 22, CZ-701 03 Ostrava, Czech Republic; michal.burda@osu.cz; 3 Department of Ecology, Faculty of Environmental Sciences, Czech University of Life Sciences Prague, Kamýcká 129, CZ-165 00 Praha-Suchdol, Czech Republic; harabis@fzp.czu.cz

**Keywords:** freshwater diversity, aquatic insects, land use conversion, biotic homogenization, bioindicators, odonata, damselfly, dragonfly

## Abstract

**Simple Summary:**

In this study, we aimed to evaluate the relationship between human transformations of land use/land cover and adult dragonfly diversity. Based on previous studies, we assumed that with increasing rates of environmental degradation and declining levels of naturalness, the representation of species with high conservation value would significantly decrease, which, however, would not affect the regional alpha diversity. Our results have shown that species richness did not correspond to habitat naturalness, but the occurrence of endangered species was significantly positively correlated with increasing naturalness; thus, habitat degradation and/or the level of naturalness significantly affected species composition, while species richness remained unchanged. Based on our analyses, it is evident that most natural areas, and therefore the least affected areas, provide suitable conditions for the largest number of endangered species. This research extends our knowledge about the impact of human activities, especially the conversion and degradation of habitats, on the composition of odonates and freshwater animals at the regional scale.

**Abstract:**

Understanding the impact of the changing proportion of land-use patterns on species diversity is a critical issue in conservation biology, and odonates are good bioindicators of these environmental changes. Some freshwater ecosystems that have been modified due to human activities can serve as important secondary habitats for odonate assemblages; however, the majority of studies addressing the value of secondary habitats in industrial and urban areas for adult dragonfly diversity have been limited to the local scale, and the value of such habitats for gamma diversity is still unclear. The aim of this study was to determine the relationship between human transformations of land use/land cover and dragonfly diversity. We interpolated the information based on dragonfly occurrence per grid cell and land cover data, indicating naturalness and degradation in 677 grid cells in the Czech Republic. Species richness did not correspond to habitat naturalness, but the occurrence of endangered species was significantly positively correlated with increasing naturalness; thus, habitat degradation and/or the level of naturalness significantly affected species composition, while species richness remained unchanged. Threatened species that occur predominantly in natural areas and threatened species with a dominant occurrence in degraded squares were also separated, which indicated that the conservation of the latter should be prioritised.

## 1. Introduction

Biodiversity faces growing pressures from urbanisation and other human activities that eliminate large portions of the habitat from the landscape. In particular, fragmentation, conversion, and degradation of habitats are considered as causes of global biodiversity decline [1,2,3], and recent evidence suggests that human-dominated areas lose significantly more biodiversity than regions where more natural habitats remain, including aquatic and terrestrial environments [3,4]. Therefore, there is no doubt that anthropogenic transformations of land cover, land use, and associated pressures strongly reduce local terrestrial biodiversity [2,3] in a wide variety of climates and environments around the world, from the tropics [5] to the polar regions [6]. However, freshwater ecosystems are far more imperiled than their terrestrial or marine counterparts [7]. Dudgeon et al. [8] identified five major threat categories to global freshwater biodiversity: overexploitation, water pollution, flow modification, and destruction or degradation of habitats, which also applies to protected areas [9]. Other important habitat changes in freshwater ecosystems include the loss of wetlands owing to drainage and other conversions from natural habitat to agriculture or urbanization [10]. Furthermore, domestic and urban pollution, agriculture (and its pollution), urbanisation, and recreational development are among the biggest threats to the biodiversity of odonates [11].

Previous studies have reported that the continued urbanisation of landscapes on a global scale currently threatens many groups of animals, both aquatic and terrestrial, such as amphibians [1,8] and birds [12,13], respectively. However, there are more problems related to community responses to large-scale disturbances and to the consequences of habitat loss and environmental degradation. Overall, the results of these studies support the view that the impacts of urbanisation on individual species do not depend only on particular sensitivities to environmental disturbances, that is, urban-adapted species may persist, whereas urban-sensitive species may not. However, it also depends on the life-history attributes of the species. Studies have shown that some species are more likely to be negatively affected by urbanisation, particularly species associated with forested habitats or species with complex life cycles, depending on landscape complexity, that is, the variety of different landscape elements to complete their life cycles [1]. Similarly, Devictor et al. [14] found that the functional diversity of communities is strongly negatively affected by landscape disturbances and land-cover changes, and the consequent habitat degradation may lead to biotic homogenisation at the global level. In line with these findings, it has been hypothesised that odonates with various life history traits (in size, dispersal ability, tolerance limits, etc.) respond differently to changes in abiotic and biotic factors. It can be expected that smaller-bodied odonates with a lower power of dispersal but with strong habitat affinity are more likely to be negatively affected by anthropogenic transformations of land use/land cover than larger-bodied odonates with higher dispersal ability. Thermal niche requirements may also play a role in this pattern. Anisopterans tend to be more abundant in open habitats with more sunlight, including habitats affected by the consequences of landscape alterations and anthropogenic disturbances, whereas zygopterans are often thermoconformers that can tolerate shaded areas, e.g., natural habitats with a complete canopy in an intact forested matrix [15].

Several studies relating to the effect of human transformations of land use/land cover on richness at a regional spatial scale and the diversity of both larval and adult odonate assemblages have been conducted. Rocha-Ortega et al. [16] conducted a cross-country analysis of the diversity of Mexican odonates and showed that land use changes affect the composition of odonates and that landscape degradation not only negatively affects habitat specialists but may also benefit odonate species with fewer specific habitat requirements. In a study that examined the species occupancy patterns in boreal forest ponds at a large spatial scale, Honkanen et al. [17] found that rare species did not seem to contribute much to variation in species richness patterns, whereas common species tended to be strongly correlated with some selected environmental variables. One of the most relevant outcomes related to the large-scale effects of anthropogenic land use on the distribution of odonates in landscapes with a long history of anthropogenic alterations was reported by Goertzen and Suhling [18], who found that the impact of urbanisation on odonate diversity is lower than that of intensive agriculture; therefore, diversity in urban landscapes is higher than in agricultural landscapes but is altered in comparison with natural landscapes.

Generally, there have been a number of studies involving odonates as bioindicators, wherein odonate assemblages were reported to be excellent groups of semiaquatic insects for environmental assessment. One of the most robust evaluation tools is the Dragonfly Biotic Index (DBI), which can be used to assess the quality of freshwater habitats at a landscape scale [19,20,21], as well as being a basic criterion for the identification of habitats with high conservation value, including secondary habitats [22,23]. Vorster et al. [24] were apparently the first to use the DBI in their comparative study (e.g., comparison of urban and rural environments) at a continental level, that is, to cover the entire African continent. In another major study at the national scale, Rocha-Ortega et al. [16] listed the main features of odonate assemblages that were used as indicators of land use intensification. Both authors reported that Anisoptera and/or large species (body size is considered a more important variable than taxonomic classification) serve as good indicators of recent land cover changes, whereas Zygoptera and/or smaller species provide information on the long-term modifications or historical effects of land use. It seems that the group of odonates can reflect the ecological status of the ecosystems at a large spatial scale much more efficiently than the use of selected species as bioindicators.

Overall, previous studies have shown that understanding how landscape patterns and their changes affect odonates’ species richness, species composition, and abundance/density has become a critical issue in conservation biology. Many studies have revealed that secondary habitats in industrial and urban areas can be seen as valuable refuges for some of Europe’s most threatened or endangered species, which were also included in the EU Habitat Directive and the Bern Convention annexes, such as *Coenagrion mercuriale* [25], *C. ornatum* [26], *Ophiogomphus cecilia* [27], and *Leucorrhinia pectoralis* [28]. However, these studies have been limited to the scale of local sites, and the evidence for this phenomenon at a regional spatial scale is inconclusive. Therefore, much uncertainty still exists about the relationship between information about the level of degradation of habitats in the landscape and the conservation value of species diversity. In addition, the manner in which regional proportions of (semi-)natural habitats affect biodiversity within the meaning of “conservation value” [29] is not widely understood.

This study aims to clarify the relationship between human transformations of land use/land cover and dragonfly diversity. Based on previous studies, we assumed that with increasing rates of environmental degradation and declining levels of naturalness, the representation of species with a high conservation value would significantly decrease, which, however, would not affect the regional alpha diversity. We also expected that with a greater degree of environmental degradation, the proportion of species from the suborder Zygoptera will decrease in favor of the suborder Anisoptera.

## 2. Materials and Methods

**Figure 1:**
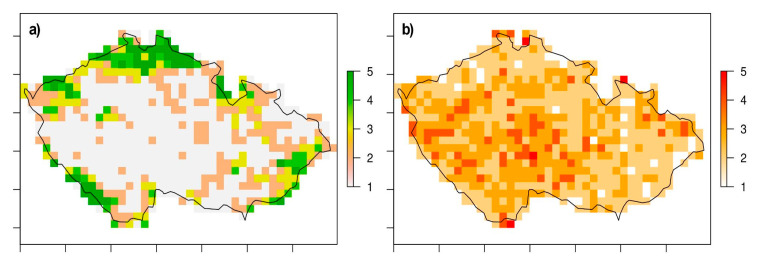
Both the (**a**) naturalness and (**b**) degradation parameters take on values from 1 (the lowest level of parameter) to 5 (the highest level of parameter).

Our analysis used two types of datasets. The first dataset contained data about the spatial distribution of areas with different coincidences of natural biotopes from Boucníková & Kučera [30]. We used their information based on the CORINE land cover data from 1990 and 2000 for naturalness, which is the proportion of natural biotopes in landscape types, and degradation, expressed as a proportion of the area covered by biotopes with low representativity and conservation status, in a grid cell of all 677 cells in the Czech Republic (Figure 1). Each grid cell, which was a rectangular spatial unit of 10′ (longitude) × 6′ (latitude), represented approximately an area of 12.0 × 11.1 km (133.2 km^2^). The second dataset contained data on the occurrence of odonates in the Czech Republic between the years 2000 and 2010 (as a suitable time period with respect to the first data), which consists of data used in the book by Dolný et al. [31] and data from the Species Occurrence Database run by the Nature Conservation Agency of the Czech Republic [32]; thus, 64,342 individual odonate records were used. These data were used without prioritizing any type of environment, so the records come from all types of aquatic and terrestrial environments within the grid cell.

**Figure 2:**
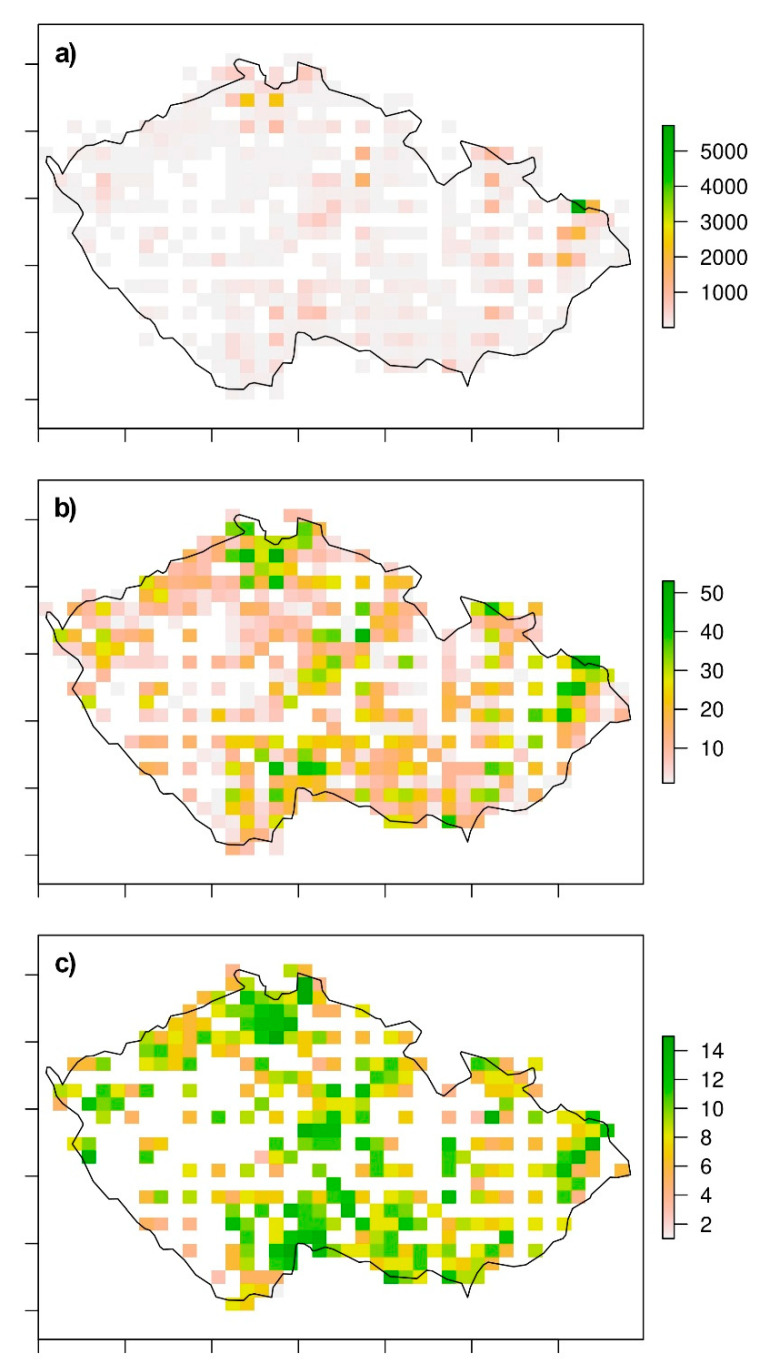
Records of odonates occurrences: (**a**) source distribution data, (**b**) source data of species richness, and (**c**) data of species richness after homogenisation.

However, the distribution of records was not balanced among all grid cells. Some of the map grid cells were assigned with a large number of reported presences of odonata species simply because of the greater sampling effort of the area, while other grid cells, usually in agricultural areas, had significantly fewer records. Such an imbalance may cause bias in species richness assessment. To overcome this problem, all data characteristics were homogenised with respect to species richness (Figure 2).

Let *c* be some desired statistical characteristics of a map grid cell based on the sum of the DBI and the sum of the threats based on the Red List of Threatened Species in the Czech Republic for the selected period [33]. We used the DBI [34] to determine the conservation value of land-use-related assemblages. This index is based on the species quality scores regarding geographic distribution in the investigated area (regional rarity), threat status/endangerment (national Red List), and species sensitivity to habitat disturbance, thereby including several main metrics of “conservation value” according to different quantification methods [29]. The subsequent sampling technique was used to homogenise *c* across all map grid cells:

A sampling threshold *t* = 15 was set in advance;

Map grid cells with less than *t* odonata presence records were excluded from further analyses.

For each map grid cell, 1000 ordinary bootstrap replicates of presence records were generated. From that, only the first *t* records were selected, and the desired statistical characteristic *c* was computed. The mean of the resulting 1000 samples of the *c* characteristic is the homogenised value of *c* for the given map grid cell. This ensures that all map grid cells obtain a comparable value of the characteristic *c* in terms of species richness because for each grid cell, only *t* presence records are considered. On the other hand, averaging the sampled values reduces the sampling error where more than *t* presence records are available.

The relationship between the analysed characteristics and degradation and naturalness was assessed using Pearson’s product moment correlation coefficient (for numeric data) or Fisher’s exact test (for count data). All data were analysed using R version 4.0.3 [35].

## 3. Results

**Figure 3:**
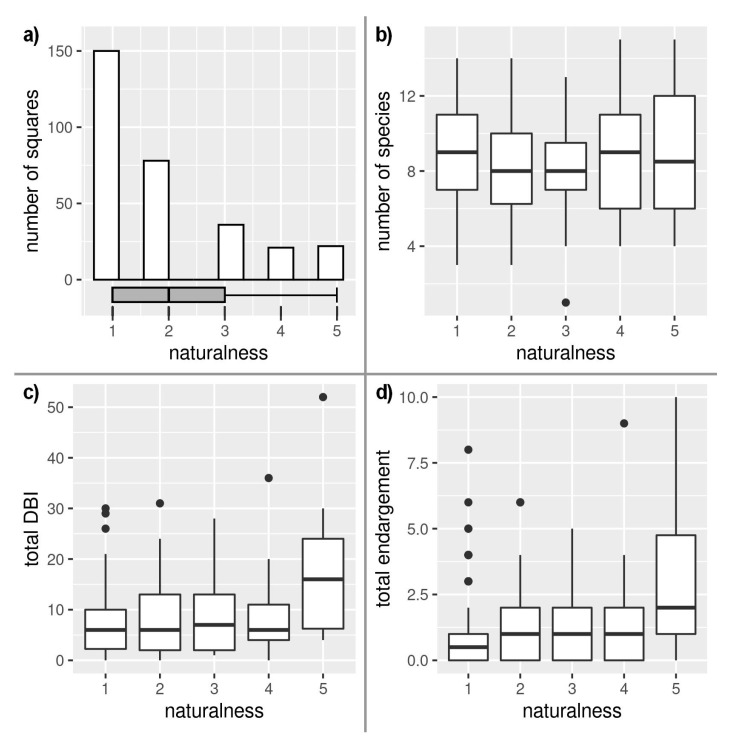
Dependence of naturalness on: (**a**) number of squares in a grid, (**b**) species richness, (**c**) Dragonfly biotic index (DBI), and (**d**) endangerment.

Based on the comparison of the occurrence in individual grid cells, it is evident that the DBI on a regional scale strongly correlates with the number of endangered species (r = 0.87, df = 305, *p* < 0.001). However, it is certainly not the case that the mean species richness in the grid cell directly predetermines the areas with the highest naturalness (Figure 3).

**Figure 4:**
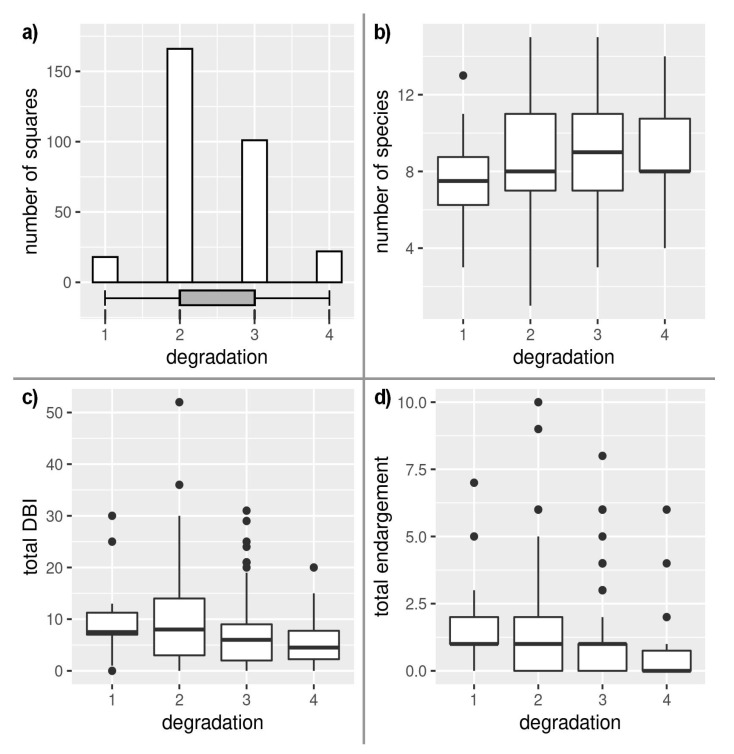
Dependence of degradation on: (**a**) number of square grids, (**b**) species richness, (**c**) Dragonfly biotic index (DBI), and (**d**) endangerment.

The territory of the Czech Republic is relatively intensively managed, while most of the territory falls into the very lowest category of naturalness (Figure 1), and the least degraded habitats are located mainly in mountainous areas (above 700 m a. s. l.), with relatively low species richness (t = 6.25, df = 22.533, *p* < 0.001), but a high proportion of endangered species significantly decreased with an increasing degree of degradation (t = −2.1977, df = 305, *p* = 0.029; Figure 4). Paradoxically, even the most degraded areas provided suitable conditions for relatively species-rich communities, but their conservation value (DBI) significantly decreased in more degraded areas (t = −3.178, df = 305, *p* = 0.002; Figure 4).

Surprisingly, the mean species richness in the grid cell did not correspond to the value of habitat naturalness, and the representation of endangered species significantly correlated with the increasing naturalness (t = 5.269, df = 305, *p* < 0.001; Figure 3). From the analysis, it is evident that the grid cells with the highest conservation values were those in areas with the highest naturalness, although the difference between the degree of naturalness 4 and 5 was not significant (*p* = 0.1154).

We also found that the degree of naturalness and degradation reflects the proportional representation of the taxa above species, i.e., the two suborders, Zygoptera (damselflies) and Anisoptera (true dragonflies), and the two largest families, Coenagrionidae (Zygoptera) and Libellulidae (Anisoptera). Suborder Anisoptera show no relation with degradation but have a positive correlation with naturalness (t = 1.962, df = 305, *p* = 0.05), mainly due to species out of the family Libellulidae (t = 2.7485, df = 305, *p* = 0.006). Suborder Zygoptera significantly correlates with both degradation (t = 2.4144, df = 305, *p* = 0.016, r = 0.1369435) and naturalness (t = −2.7645, df = 305, *p* = 0.006) with different trends. Increasing levels of degradation co-occurred with an increasing number of species of the family Coenagrionidae (t = 2.2154, df = 305, *p* = 0.027). On the other hand, increasing levels of naturalness were associated with a decreasing number of species outside of the family Coenagrionidae (t = −3.4364, df = 305, *p* < 0.001).

Only a few species reflected habitat degradation from the perspective of a particular species (Table 1), while only two species, *Coenagrion lunulatum* and *C. hastulatum*, represented habitat specialists. Based on the evaluation of the occurrence of individual species in relation to naturalness, it was possible to identify several groups of species. The largest group consisted of species associated with different types of peat habitats with a high degree of naturalness. These groups include endangered species, such as *Aeshna caerulea*, *A. subarctica*, *Somatochlora alpestris*, *S. arctica*, or *Leucorrhinia rubicunda*, but also the habitat of specialists that do not belong to the endangered species, namely, *A. juncea*, *C. hastulatum*, *L. dubia*, and *Sympetrum danae*. The second group are species that are relatively numerous even in areas with a lower value of naturalness; however, in areas with higher values of naturalness, they are significantly more frequent. This includes, for example, *A. cyanea*, *Cordulia aenea*, *Enallagma cyathigerum*, *Pyrrhosoma nymphula*, and *Orthetrum coerulescens*. *Cordulegaster bidentata* is associated with small streams in areas of highly preserved forests. An essential group are species such as *Sympecma paedisca* and *Sympetrum pedemontanum*, which, although they show a certain correlation with naturalness, appear in localities with high and low degrees of naturalness. The same category can also include species like *Sympetrum depressiusculum* and *Coenagrion ornatum*, which are among the most endangered species in Europe, but in the Czech Republic, they occur mainly in areas with a lower degree of naturalness.

The last group includes expanding thermophilic species such as *Anax parthenope*, *Crocothemis erythrea*, and *Orthetrum albistylum*, which occur in habitats with lower naturalness.

## 4. Discussion

This research extends our knowledge about the impact of human activities, especially the conversion and degradation of habitats, on the composition of odonates and freshwater animals at the regional scale. In summary, these results show that habitat degradation and/or the level of naturalness had significantly stronger effects on species composition than on species richness. Human activity and habitat fragmentation and degradation at the landscape scale did not alter species richness in dragonfly assemblages but led to large changes in species composition, that is, the proportion of habitat specialists (late-successional specialists replaced with generalists) and the proportion of species of local conservation concern or threatened species declined. This pattern suggests that the natural and degraded landscapes would support maximal regional species richness but would lead to taxonomic homogenisation and functional homogenisation at a regional scale.

Based on our analyses, it is evident that most natural areas, and therefore the least affected areas, provide suitable conditions for the largest number of endangered species. This is very important, especially for species associated with certain types of climax habitats, such as raised bogs, fens, and some other types of late successional habitats. These species are among the species with the most negative trends in occurrence [36]. The main causes of the decline of these species are not only the degradation of natural habitats but also desiccation and other negative changes associated with global climate change [3,18].

As expected, our results demonstrate that the taxa above species (suborders, families), e.g., zygopterans and anisopterans, responded in different ways to changes in the environment, including to land use intensification [16]. Nevertheless, the findings are in partial contradiction with these previous results reported in the literature, especially the fact that anisopterans show a positive correlation with naturalness. This probably reflects the fact that the family-level approach is more accurate than a simple, suborder level-based approach in the bioindication of anthropogenic impacts on ecosystems [37]. Our results correlate well with that study and further support the concept that the proportion of Libellulidae/other Anisoptera significantly increase along a disturbance gradient. On the other hand, in contradiction with the earlier findings of Šigutová et al. [37], the observations of our research indicate a decrease in the proportions of Coenagrionidae/other Zygoptera with higher levels of naturalness. The observed decreasing number of species of the non-Coenagrionidae zygopterans with an increasing level of naturalness could be interpreted as being a result of the effects of rising altitude. Pristine and natural habitats are most common in the mountainous and high elevation regions of the Czech Republic, while the vast majority of non-zygopterans (e.g., Lestidae species) are most commonly found in lowland habitats and appears to prefer low elevations with moderate temperatures. In sum, some of these observations are unexpected, and these data thus need to be interpreted with caution.

In contrast, our analysis identified species such as *L. pectoralis* or *O. cecilia*, which enjoy great attention to nature conservation, and their even distribution in areas of different levels of naturalness indicates that these species are able to adapt to anthropogenically altered areas. However, the frequent occurrence in less natural areas may not necessarily be related only to the ecological valence of the species and its ability to tolerate anthropogenic influences but may be associated with a very limited supply of natural habitats of certain types [38]. The findings of the current study are consistent with those of Iversen et al. [39], who examined the distribution and breeding habitat choices of *L. pectoralis* in their study of national changes in land use influence in Estonia. *Leucorrhinia pectoralis* was found almost as much in the unrestored landscapes as in the restored landscape (43 % and 51 %, respectively), and the occupation frequency confirmed that the species was not significantly more abundant in one landscape type than in the other. Our results are also consistent with those of other studies, which suggest that cities may have the potential to host species of conservation concern and allocate permanent habitats for *O. cecilia*, as well as for *L. pectoralis* [18,27].

On the other hand, 12 % of all odonate species occurring in Europe are included in the Annexes of the EU Habitats Directive, which indicates that odonates are a highly prioritised group compared with other groups of invertebrates. However, this fact does not reflect the current situation because 19 of the 22 threatened species on the Red List are not among the priority species included in the EU Habitats Directive [40]. The EU Habitats Directive, adopted in 1992, sets out national actions to protect and prioritise endangered and endemic European species, including odonates [41]. Nevertheless, the distribution of species listed in the appendices of the EU Habitats Directive is geographically biased towards the species occurring in Central and Western European countries [42]. South European species with often very small areas, which are often endangered by immediate extinction, are often not among the priority targets of species protection [42,43]. However, this should not be the case. In addition, among West and Central European species, we can find several that deserve more attention. Almost 30 years after its implementation, the Habitats Directive has not been updated, and the need for effective measures for certain species is more important than ever. Freshwater ecosystems currently face a cocktail of anthropogenic influences, and it is often very difficult to determine the main cause of threats to a given taxon [8,44]. In addition, odonates strongly reflect the state of the surrounding terrestrial environment [23,45]. Therefore, it seems very effective to focus conservation measures on most areas that are least affected by human impact, as recent or past anthropogenic changes do not affect all habitat types and species that occur in each of these habitats with the same intensity. In Central Europe, we identified several species in need of conservation attention, which have in common only the fact that they often occur in secondary habitats. For example, *C. ornatum*, a species that has been included in the Annexes of the EU Habitats Directive: in many regions, we simply cannot find a single locality of this species with a higher degree of naturalness [26]. Another example is *S. depressiusculum*. Although this species often forms large populations and is able to use a wider range of habitats, it is declining throughout Europe and is included in the European Red List [11]. Nevertheless, for example, in the Czech Republic, we only have the last population of this species, which hovers above the extinction vortex. This is because the occurrence of *S. depressiusculum* is associated with the natural dynamics of watercourses, and larger populations in Central Europe are known primarily from fry ponds, that is, secondary habitats with a low degree of naturalness [46,47].

Based on our analysis, it is clear that other species regarded as threatened, such as *S. pedemontanum* or *S. paedisca*, are comparable to those mentioned in the previous statements. They occur in areas with a high degree of naturalness, as well as in areas with highly anthropogenically affected areas. *Sympecma paedisca* is one of the species in the Annexes of the EU Habitats Directive, but it belongs to the non-priority species.

A variety of responses were elicited in response to the question, “Is it possible to protect long-term even a species whose natural habitat has completely disappeared?” Experience from the Netherlands shows that negative trends in dragonfly distribution can be reversed, and many populations can be recovered [48]. On the other hand, the options for species conservation outside areas with a high degree of naturalness are limited. The reason is not only the degradation of the environment but also certain limits of nature conservation, which still have not been overcome. The protection of species outside untouched areas requires active management, which is often very costly and must be targeted very precisely to be effective. Cardoso et al. [49] identified seven impediments to effective invertebrate species protection, the (non-)solutions of which significantly reduced the chances of individual species being saved from extinction, among them: (1) the distribution of invertebrates is mostly unknown, (2) the abundance of species and their changes in space and time are unknown, and (3) species ways of life and sensitivities to habitat change are largely unknown.

Dragonflies and damselflies are among the most popular, and therefore the best known, insect groups. Nevertheless, even with Central European species, we encounter the fact that for a relatively large proportion of species, we do not even know population trends [40]. We still have very limited information about their ecology and their sensitivity to habitat change (a problem known as the Hutchinsonian shortfall), and we can hardly reveal the true cause of their decline. Cardoso et al. [49] identified good indicator taxa and studied extinction rates using indirect evidence. As an example, in the Czech Republic, we have only been able to discuss systematic monitoring in the last 15 years, so we lack comparable data for the evaluation of long-term trends. To separate rare species from endangered species, we can only assess the current distribution and other indirect evidence. One of the indirect indicators may be naturalness; more precisely, information on whether the majority populations of a given species occur in the natural environment (e.g., species associated with peat bogs) or even a very rare species occurring mainly in secondary habitats in areas with a low degree of naturalness. Although there are examples of secondary habitats providing suitable conditions for rare and endangered species [22], their extinction is certainly far more likely than in natural habitats [38,50].

## 5. Conclusions

In general, it is far more effective to protect species in their natural environment than to create entirely new habitats. The question is how to protect the species in a situation where there are simply no natural alternatives. Anthropogenically induced changes in the environment act selectively, while certain types of habitats are altered/degraded with much greater intensity. In addition, in the recent past, processes that have significantly contributed to the natural regeneration of freshwater ecosystems have been significantly slowed down. Based on our analysis, it is evident that populations of several rare species persist in heavily anthropogenically altered habitats. For many species, however, this is probably not due to their ability to adapt to new conditions but rather the degradation of all natural alternatives. Conservation of these species has reached the point where the only option is management in the form of creating new suitable habitats.

## Figures and Tables

**Table 1:** Information on the Dragonfly Biotic Index (DBI) value, the degree of endangerment for the observed period, and the dependence on degradation and naturalness were assessed using Fisher’s test for the individual species of odonates from the Czech Republic (CR) included in the study. Species are sorted according to the dependency rate on naturalness.

Species	DBI	Red List CR (2005)	Degradation (*p*-Value)	Naturalness (*p*-Value)
*Aeshna juncea* (Linnaeus, 1758)	4	VU	0.9338	<0.0001
*Leucorrhinia dubia* (Vander Linden, 1825)	6	VU	0.9167	<0.0001
*Somatochlora alpestris* (Selys, 1840)	7	EN	0.0867	<0.0001
*Somatochlora arctica* (Zetterstedt, 1840)	7	EN	0.5130	<0.0001
*Cordulegaster bidentata* (Selys, 1843)	5	VU	0.0728	0.0001
*Sympetrum danae* (Sulzer, 1776)	1		0.4139	0.0004
*Aeshna caerulea* (Ström, 1783)	9	CR	0.3008	0.0006
*Aeshna cyanea* (Müller, 1764)	0		0.9445	0.0017
*Platycnemis pennipes* (Pallas, 1771)	0		0.7900	0.0036
*Cordulegaster boltonii* (Donovan, 1807)	3	VU	0.2165	0.0066
*Aeshna subarctica* (Walker, 1908)	9	CR	0.6880	0.0083
*Leucorrhinia rubicunda* (Linnaeus, 1758)	7	EN	0.4556	0.0106
*Pyrrhosoma nymphula* (Sulzer, 1776)	0		0.4084	0.0109
*Ischnura elegans* (Vander Linden, 1820)	0		0.5523	0.0147
*Sympecma paedisca* (Brauer, 1877)	6	CR	0.8281	0.0215
*Coenagrion hastulatum* (Charpentier, 1825)	4	NT	0.0050	0.0243
*Orthetrum coerulescens* (Fabricius, 1798)	5	EN	0.8589	0.0299
*Enallagma cyathigerum* (Charpentier, 1840)	0		0.0211	0.0302
*Crocothemis erythraea* (Brullé, 1832)	1		0.4823	0.0330
*Sympetrum pedemontanum* (Müller in Allioni, 1766)	7	EN	0.7181	0.0471
*Orthetrum albistylum* (Selys, 1848)	1		0.3890	0.0486
*Anax parthenope* (Selys, 1839)	1	VU	0.2992	0.0505
*Lestes barbarus* (Fabricius, 1798)	5	VU	0.5384	0.1180
*Cordulia aenea* (Linnaeus, 1758)	0		0.4309	0.1276
*Coenagrion ornatum* (Selys, 1850)	7	CR	0.6649	0.1474
*Somatochlora metallica* (Vander Linden, 1825)	0		0.0315	0.1700
*Anax imperator* (Leach in Brewster, 1815)	0		0.1206	0.1828
*Somatochlora flavomaculata* (Vander Linden, 1825)	6	EN	0.6152	0.2008
*Libellula quadrimaculata* (Linnaeus, 1758)	0		0.2112	0.2205
*Lestes sponsa* (Hansemann, 1823)	0		0.0040	0.2346
*Leucorrhinia pectoralis* (Charpentier, 1825)	5	VU	0.6519	0.2407
*Erythromma lindenii* (Selys, 1840)	6		1.0000	0.2573
*Calopteryx virgo* (Linnaeus, 1758)	1		0.3937	0.2908
*Erythromma najas* (Hansemann, 1823)	1		0.0942	0.3338
*Leucorrhinia albifrons* (Burmeister, 1839)	8	CR	0.7565	0.3802
*Brachytron pratense* (Müller, 1764)	5	EN	0.1447	0.3869
*Coenagrion puella* (Linnaeus, 1758)	0		0.3854	0.3994
*Aeshna grandis* (Linnaeus, 1758)	1		0.0219	0.4091
*Ophiogomphus cecilia* (Geoffroy in Fourcroy, 1785)	4	EN	0.1385	0.4137
*Erythromma viridulum* (Charpentier, 1840)	1	NT	0.7449	0.4604
*Sympetrum striolatum* (Charpentier, 1840)	2	NT	0.8467	0.4790
*Calopteryx splendens* (Harris, 1780)	0		0.3071	0.4797
*Orthetrum cancellatum* (Linnaeus, 1758)	0		0.5501	0.4910
*Nehalennia speciosa* (Charpentier, 1840)	9	CR	0.4593	0.5114
*Chalcolestes viridis* (Vander Linden, 1820)	1		0.4308	0.5149
*Gomphus vulgatissimus* (Linnaeus, 1758)	2	VU	0.1256	0.5161
*Coenagrion lunulatum* (Charpentier, 1840)	9	CR	0.0340	0.5713
*Epitheca bimaculata* (Charpentier, 1825)	8	CR	0.7103	0.6077
*Libellula depressa* (Linnaeus, 1758)	0		0.2256	0.6144
*Sympetrum meridionale* (Selys, 1841)	5	EN	1.0000	0.6435
*Sympetrum fonscolombii* (Selys, 1840)	3	EN	0.6530	0.6462
*Aeshna affinis* (Vander Linden, 1820)	3	VU	1.0000	0.6575
*Stylurus flavipes* (Charpentier, 1825)	6	EN	0.8271	0.6630
*Aeshna isoceles* (Müller, 1767),	4	VU	0.6342	0.7061
*Aeshna mixta* (Latreille, 1805)	1		0.4712	0.7132
*Lestes virens* (Charpentier, 1825)	2	VU	0.8623	0.7134
*Orthetrum brunneum* (Fonscolombe, 1837)	4	EN	0.6302	0.7219
*Onychogomphus forcipatus* (Linnaeus, 1758)	5	EN	0.4745	0.7235
*Sympetrum sanguineum* (Müller, 1764)	0		0.5376	0.7354
*Coenagrion pulchellum* (Vander Linden, 1825)	3		0.8519	0.7732
*Sympecma fusca* (Vander Linden, 1820)	1	NT	0.2797	0.7989
*Sympetrum depressiusculum* (Selys, 1841)	9	CR	0.7883	0.8177
*Sympetrum flaveolum* (Linnaeus, 1758)	4		0.3339	0.8472
*Lestes dryas* (Kirby, 1890)	5	VU	0.3553	0.8598
*Ischnura pumilio* (Charpentier, 1825)	3	NT	0.8598	0.8622
*Sympetrum vulgatum* (Linnaeus, 1758)	0		0.2521	0.8780
*Libellula fulva* (Müller, 1764)	6	CR	0.4937	0.9696
*Coenagrion scitulum* (Rambur, 1842)	5	CR	0.9077	1.0000

## Data Availability

Derived data supporting the findings of this study are available from the corresponding author on request.
